# Feasibility of Quantifying Arterial Cerebral Blood Volume Using Multiphase Alternate Ascending/Descending Directional Navigation (ALADDIN)

**DOI:** 10.1371/journal.pone.0156687

**Published:** 2016-06-03

**Authors:** Ki Hwan Kim, Seung Hong Choi, Sung-Hong Park

**Affiliations:** 1 Graduate School of Medical Science and Engineering, Korea Advanced Institute of Science and Technology, Daejeon, South Korea; 2 Department of Radiology, Seoul National University College of Medicine, Seoul, South Korea; 3 Department of Bio and Brain Engineering, Korea Advanced Institute of Science and Technology, Daejeon, South Korea; INSERM U894, FRANCE

## Abstract

Arterial cerebral blood volume (aCBV) is associated with many physiologic and pathologic conditions. Recently, multiphase balanced steady state free precession (bSSFP) readout was introduced to measure labeled blood signals in the arterial compartment, based on the fact that signal difference between labeled and unlabeled blood decreases with the number of RF pulses that is affected by blood velocity. In this study, we evaluated the feasibility of a new 2D inter-slice bSSFP-based arterial spin labeling (ASL) technique termed, alternate ascending/descending directional navigation (ALADDIN), to quantify aCBV using multiphase acquisition in six healthy subjects. A new kinetic model considering bSSFP RF perturbations was proposed to describe the multiphase data and thus to quantify aCBV. Since the inter-slice time delay (TD) and gap affected the distribution of labeled blood spins in the arterial and tissue compartments, we performed the experiments with two TDs (0 and 500 ms) and two gaps (300% and 450% of slice thickness) to evaluate their roles in quantifying aCBV. Comparison studies using our technique and an existing method termed arterial volume using arterial spin tagging (AVAST) were also separately performed in five subjects. At 300% gap or 500-ms TD, significant tissue perfusion signals were demonstrated, while tissue perfusion signals were minimized and arterial signals were maximized at 450% gap and 0-ms TD. ALADDIN has an advantage of visualizing bi-directional flow effects (ascending/descending) in a single experiment. Labeling efficiency (α) of inter-slice blood flow effects could be measured in the superior sagittal sinus (SSS) (20.8±3.7%.) and was used for aCBV quantification. As a result of fitting to the proposed model, aCBV values in gray matter (1.4–2.3 mL/100 mL) were in good agreement with those from literature. Our technique showed high correlation with AVAST, especially when arterial signals were accentuated (i.e., when TD = 0 ms) (r = 0.53). The bi-directional perfusion imaging with multiphase ALADDIN approach can be an alternative to existing techniques for quantification of aCBV.

## Introduction

Magnetic resonance imaging (MRI) has been widely used to quantify cerebral hemodynamics. Dynamic susceptibility contrast MRI (DSC-MRI) is a conventional perfusion-weighted imaging technique that measures the T_2_* signal decrease during the first passage of an intravascular contrast agent through the cerebral vasculature [1]. Another technique is arterial spin labeling (ASL), which uses magnetically labeled blood water protons as an endogenous tracer [2]. ASL has been utilized to quantify the perfusion in the brain and other organs for the last two decades and is suitable for healthy subjects, patients with renal disease, and pediatric patients due to its noninvasiveness. Although brain perfusion has been investigated in many perfusion quantification studies, most techniques have focused on measurement of tissue perfusions.

Brain hemodynamics is controlled by many factors including neuronal activity, metabolic demand, and carbon dioxide concentration. Arterial blood flow/volume is closely related to regulation of these factors [3]. The ability of vasoconstriction and vasodilatation of arteries controls brain hemodynamics and acts as an indicator for the vascular reserve [4]. Furthermore, arterial cerebral blood volume (aCBV) is proven to be related to response of the neuronal stimulations with short stimulation duration [5–7]. Therefore, the evaluation of aCBV is meaningful in many clinical research and neuroscience applications.

Several techniques have been proposed for measurement of aCBV by ASL such as (i) selective suppression of blood signals [8–11], (ii) separation based on oxygen level [12], (iii) pseudo-diffusion coefficient acquired by intravoxel incoherent motion (IVIM) model [13], (iv) separation of tissue signal from blood using modulation of tissue signals by magnetic transfer (MT) effects [14], (v) separation of arterial compartment using adjustment of post-inversion delay and tagging duration [15], and (vi) multiphase balanced steady state free precession (bSSFP) readout [16]. These techniques have different limitations such as single slice imaging [10], no available data in human study [12, 14], lack of quantification [16], and need of calibration scans separately [15]. Such limitations hinder clinical translation, and there is still much room for improvement.

In multiphase bSSFP approach, a long train of radiofrequency (RF) pulses drives both labeled and unlabeled blood spins into steady state during multiphase acquisition, therefore, magnetization differences between the labeled and unlabeled spins decrease with the number of RF pulses. Because the number of RF pulses is related to the velocity and traveling distance of blood within the imaging slice, multiphase bSSFP readout provides different sensitivity to spins with different velocities. As the mean blood velocity in capillaries with 2–5 μm in diameter is ~0.8 mm/s [17], capillary blood is driven into steady state irrespective of the initial longitudinal magnetization (labeled or control). On the other hand, average blood velocity in pial arteries with a range of diameter from 20 to 200 μm is 13−60 mm/s [18]. Arterial blood has more chances to quickly move out of the imaging slice or traverse into the capillary, and thus arterial blood experiences smaller number of RF pulses. Therefore, the labeled blood in the arterial compartment is highlighted, while the labeled blood in the tissue and capillary compartments is suppressed by a long train of RF pulses [18]. When this contrast mechanism is combined with ASL technique, images mostly from arterial compartment can be acquired [16].

A sequential acquisition of 2D slices is subject to inter-slice blood flow effects on subsequent slices, because blood is continuously saturated by RF pulses during scanning. [19]. If the direction of blood flow is the same as that of acquisition order, saturated blood in prior slices can be detected in an imaging slice. A new imaging technique termed, alternate ascending/descending directional navigation (ALADDIN) was recently introduced for the acquisition of perfusion-weighted imaging, based on the inter-slice blood flow effects [20–22]. ALADDIN uses bSSFP as a readout, because of its high flip angle within a short repetition time (TR), high temporal resolution, and good sensitivity to the initial magnetization difference [23–26]. When multiphase acquisition strategy is implemented in ALADDIN, it can be an alternative technique for quantifying aCBV.

The aim of this study was to demonstrate the feasibility of quantifying aCBV using multiphase bSSFP with the ALADDIN scheme. A kinetic model was developed to describe labeled blood water signals in the multiphase bSSFP readout, and the acquired multiphase data was fitted to the kinetic model to obtain aCBV. Based on the fact that ALADDIN has an advantage of visualizing bi-directional flow effects (ascending/descending) in a single experiment, labeled blood water signals in the superior sagittal sinus (SSS) were also analyzed to estimate the labeling efficiency of ALADDIN.

## Materials and Methods

### Theory

**Fig 1:**
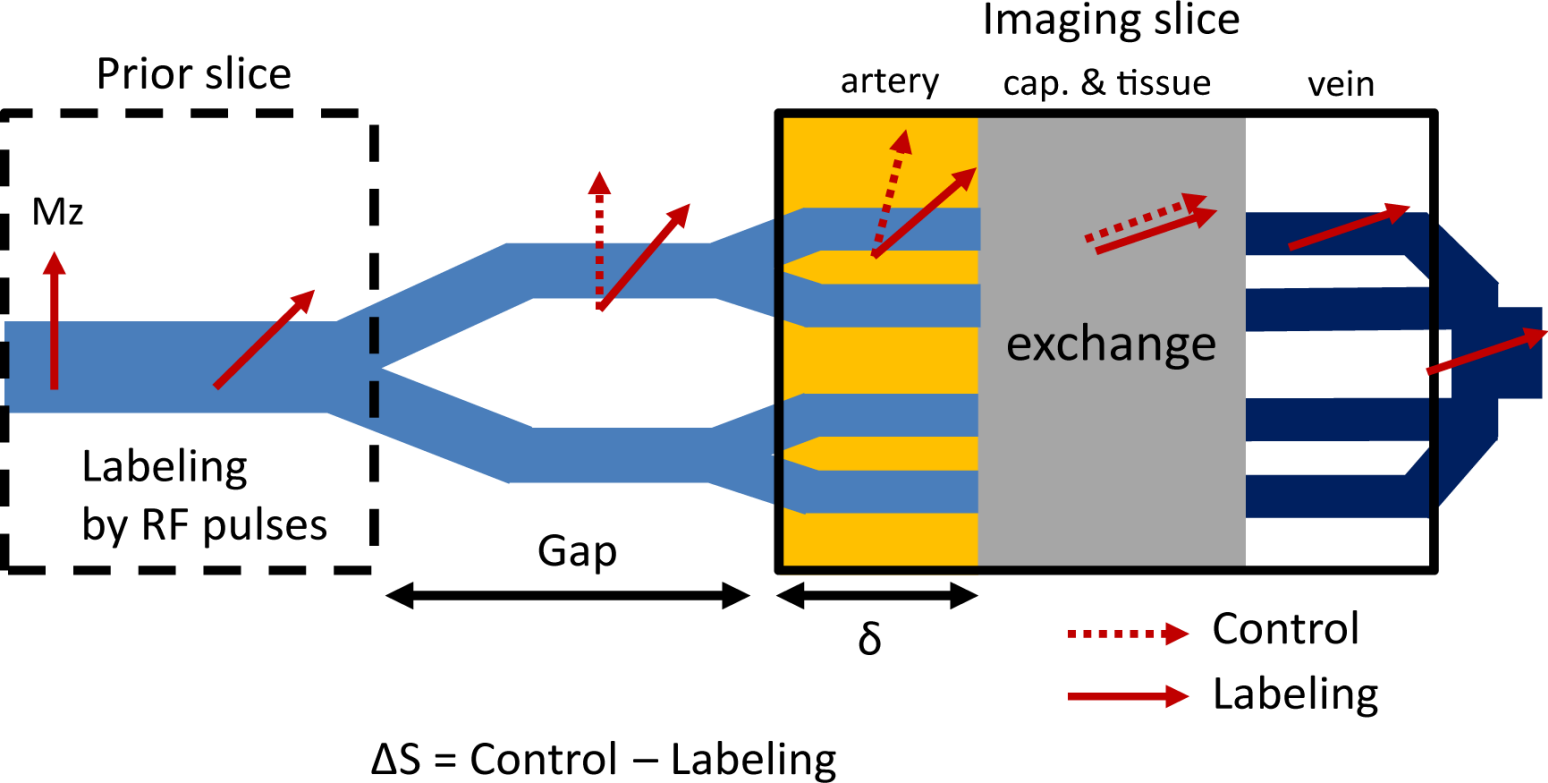
Schematic diagram for ALADDIN with multiphase bSSFP readout. Diagram demonstrates magnetization changes in the labeling and imaging slices and also in the arterial and capillary/tissue compartments. δ represents the transit time from the arterial compartment to the capillary/tissue compartment in the imaging slice. See the main text for detailed information.

The magnetization changes in the labeling and imaging planes and also in the arterial and capillary/tissue compartments are schematically demonstrated in Fig 1. In ascending order, arterial blood (solid arrows) is saturated in a prior slice (dashed box) due to excitation RF pulses (labeling). In contrast, arterial blood in descending order (dotted arrows) has full magnetization at the imaging slice (control). In case of multiphase ALADDIN, a long train of RF pulses saturate blood spins running through the imaging slice. During the transit time from the arterial compartment to the capillary/tissue compartment in the imaging slice (δ in Fig 1), both labeled and unlabeled blood spins experience smaller number of RF pulses due to high velocity and short δ. However, the longitudinal magnetizations of labeled and unlabeled blood in capillaries and tissue are driven into steady state due to nearly zero velocity. The multiphase bSSFP readout, therefore, highlights the differences in the longitudinal magnetizations of arterial blood (ΔMz) and suppresses those in the other compartments. Venous blood from tissue and capillary can be also saturated in the prior slice, and thus the reduced magnetization of venous blood can be detected in SSS in subsequent slices during descending acquisition.

Since each imaging slice acts as a labeling plane for subsequent slices, total labeled blood signals are determined by both the sequential labeling effects of all prior slices and T_1_ recovery effects, as described later. The labeling effect from the first prior slice is dominant due to the long readout time (~1.2 sec) and T_1_ recovery effects. The general kinetic model for ASL [27, 28] can be served as one of the possible models for multiphase ALADDIN. By modifying arterial input function (AIF) of the general kinetic model specific to ALADDIN, the local concentration of labeled blood spins C(t) from all prior slices can be represented as follows:

$$C(t)=2\alpha M_{0,b}\cdot F\cdot \exp(-\mathrm{ATT}/T_{1,b})\cdot unit(ATT-t)$$ (1)

where t = 0 is defined as the beginning of each slice acquisition, α is the labeling efficiency, ATT is the traveling time from a labeling plane to an imaging slice, T_1,b_ is the T_1_ relaxation time of blood, M_0,b_ is the equilibrium magnetization of blood, and *unit* is a discontinuous function whose value is zero for negative argument and one for positive argument. According to the previous simulation study [20, 22], the labeling efficiency in ALADDIN does not change significantly within the range for velocity in arteries, when blood spins with even and odd numbers of RF excitations and those with off-resonance states were averaged. Therefore, the labeling efficiency may be affected less by imaging direction, blood velocity, or vascular structures. In this study, the labeling efficiency is separately measured in SSS, as described later. Most labeled blood signals for multiphase acquisition originate from the arterial compartment, except the initial phase where both arterial and tissue compartments contribute together. The flow parameter F is in the unit of blood volume per one minute per one hundred milliliter of voxel volume (rather than the supplied tissue volume). The F value can reflect blood flow from both arterial and tissue compartments, but the tissue component is suppressed by consecutive bSSFP RF excitations.

Labeled blood signals in multiphase bSSFP can be eliminated by three factors; (i) excretion of labeled blood from an imaging voxel, (ii) T_1_ recovery of labeled blood, i.e., m(t) = exp(−t/*T*_1,*b*_), and (iii) driving into the steady state condition by a long train of RF pulses in the imaging slice. The excretion of the labeled blood can be described by a first-order exponential function, termed residue function in the general kinetic model [27, 29]. We assumed a residue function R(t) to be a first-order exponential function with transit time (δ) which is needed for blood to traverse the arterial compartment before reaching the capillaries, i.e., R(t) = exp(−t/δ).

For simplicity, let’s ignore the loss of labeled blood signals by a long train of bSSFP RF pulses and take into account only T_1_ recovery m(t) and excretion R(t) from a voxel, which is termed as T_1_ model. From Eq (1), the T_1_ model can be described by:

$$\begin{array}{l} \frac{{\Delta S}_{\left(t\right)}}{S_{0,b}}≅\frac{{\Delta M}_{b(t)}}{M_{0,b}}=2\alpha \cdot F\cdot e^{-ATT/T_{1,b}}\cdot unit(ATT-t)*(R(t)\cdot m(t))\\ \;=\left\{ \begin{array}{l} 2\alpha \cdot F\cdot \frac{\delta \cdot T_{1,b}}{T_{1,b}+\delta }\cdot e^{-ATT/T_{1,b}}\;0\leq t\leq \mathrm{ATT}\\ 2\alpha \cdot F\cdot \frac{\delta \cdot T_{1,b}}{T_{1,b}+\delta }\cdot e^{-ATT/T_{1,b}}\cdot e^{-(t-ATT)\cdot (T_{1,b}+\delta )/(\delta \cdot T_{1,b})}\;\mathrm{ATT}<t \end{array}\right. \end{array}$$ (2)

In reality, however, multiphase bSSFP readout can reduce the signal difference between labeled and unlabeled blood water. The loss of labeling effects is determined by the number of RF pulses affecting labeled blood spins in an imaging slice (*n*), which can be approximated as δ divided by the repetition time (TR). On the transient state of bSSFP, the transverse magnetization of blood M_b(n)_ is given by [30]:

$$M_{b\;(n)}=(\sin\left(FA/2\right)M_{0,b}-M_{ss,b})\rho^{n}+M_{ss,b}$$ (3)

where FA is the flip angle, M_ss,b_ is the steady state magnetization of blood [30], and ρ is the decay rate in bSSFP; ρ = exp(−TR/T_2,b_)·sin^2^(FA/2)+ exp(−TR/T_1,b_)·cos^2^(FA/2). Although labeled blood signals are reduced by T_1_ recovery before entering an imaging slice, the signal difference between labeled and unlabeled blood decreases following the bSSFP response of ρ^*n*^ described in Eq (3) during the image acquisition. The signal difference ΔS(t) between control and labeling images is calculated by the bSSFP model, which is the sum of two terms; the first term is related to T_1_ recovery m(t) occurring before arrival of labeled blood at the imaging slice and the second term describes magnetization changes caused by bSSFP response of ρ^*n*^ during the image acquisition. For the bSSFP model, the AIF shown in Eq (1) should be separated into two terms as follows.

$$C(t)=2\alpha M_{0,b}\cdot F\cdot \exp(-\mathrm{ATT}/T_{1,b})\cdot \{unit(-t)+rect\;\left(\frac{t}{ATT}-\frac{1}{2}\right)\}$$ (4)

The bSSFP model can then be described as follows.

$$\frac{{\Delta S}_{\left(t\right)}}{S_{0,b}}≅\frac{{\Delta M}_{b(t)}}{M_{0,b}}=2\alpha \cdot F\cdot e^{-\frac{ATT}{T_{1,b}}}\cdot \{(unit(-t)*(R(t)\cdot m(t)))∙\rho^{n}+rect\left(\frac{t}{ATT}-\frac{1}{2}\right)\;*\;(R(t)\cdot \rho^{n})\}$$ (5)

where *rect* is a function that is zero outside the interval [−1/2, 1/2] and one inside it.

The measurement of the baseline signal intensity in arterial vessels S_0,b_ is difficult due to limited spatial resolution. Therefore S_0,b_ can be replaced by venous signal intensity measured from SSS, which is the only vessel large enough to avoid partial volume effects [31]. During multiphase acquisition, venous blood in the imaging slice is continuously replaced by inflowing blood with equilibrium magnetization and each blood spin is affected by less than 15 RF pulses, which is calculated by slice thickness (8 mm) and mean velocity in SSS (13 cm/s) [32]. Although there is difference between T_2_ values of artery and vein, less than 15 RF pulses are not expected to make a significant difference in signal intensity based on simulations (data not shown). Therefore, it is reasonable that S_0,b_ is replaced by mean signal intensity of SSS in images of ascending order, which is free from labeling effects due to the venous flow opposite to the ascending acquisition order. By fitting the multiphase bSSFP data to the models (either Eqs (2) or (5)), we can estimate three unknown parameters (F, δ, and ATT) and thus calculate aCBV using the estimated F and δ, as follows [10, 33]:

aCBV=F∙δ (6)

#### Superior sagittal sinus

In case of ALADDIN, descending venous blood flows (image of “head ➔ feet”) can be demonstrated by subtracting images of descending order (= labeling) from those of ascending order (= control) [20]. Distinct from labeling mechanism on the arterial side, labeling mechanism on the venous side can be separated into two cases of ‘labeling from vein’ and ‘labeling from tissue’. Labeling from vein is caused by the inter-slice flow effects. The number of RF pulses applied to blood (*n*) is determined by the flow velocity (ν) in SSS (normal velocity: 13 cm/s) [32]. The transverse magnetization of blood M_b(n)_ can be calculated by Eq (3), which depends upon *n*. On the other hand, labeling from tissue can be explained by the steady state magnetization of tissue in the prior slices. During prior slice acquisition, the extravascular water spins are exposed to 288 RF pulses and reach the steady state condition. As cortical vascular mean transit time is about 3 sec as measured in dynamic susceptibility MRI [34], capillary blood in the steady state condition flows into vein. This labeling mechanism is termed labeling from tissue. The magnetization of venous blood M_b,in_ can be estimated from the steady state magnetization of tissue M_ss,t_ and the brain-blood partition coefficient λ; M_b,in_ = M_ss,t_ /λ.

Due to high flow rate, venous blood affected by labeling from vein quickly moves out of an imaging slice and can be detected only in the early phase(s) of the images. In contrast, effects of labeling from tissue decrease with T_1_ relaxation and can remain until the later phases of the images. The signal difference ΔS_b,(t)_ in SSS between the images with ascending and descending acquisition orders can be calculated, as follows.

$$\frac{{\Delta S}_{b(t)}}{S_{0,b}}≅\frac{{\Delta M}_{b(t)}}{M_{0,b}}=\left\{ \begin{array}{l} \frac{{M_{0,b}\sin\left(FA/2\right)-M}_{b\;(n)}}{M_{0,b}}∙e^{-t/T_{1,b}}\;t\leq \varepsilon \\ \frac{{M_{0,b}\sin\left(FA/2\right)-M}_{b,in}}{M_{0,b}}∙e^{-t/T_{1,b}}\;\varepsilon <t \end{array}\right.$$ (7)

where ε is the transit time of blood affected by labeling from vein.

### Simulations

#### Quantification of aCBV

In order to represent capillary and artery, simulations were performed with two flow rates of 0.2 and 5 cm/s, respectively. We compared the labeled blood signals of the bSSFP model (Eq (5)) with those of the T_1_ model (Eq (2)). The time t = 0 was defined as the beginning of image acquisition of each slice. Labeled blood may arrive at the imaging slice before, during, or after the onset of acquisition, and such time difference between the onset of image acquisition and the latest arrival of the labeled blood is dependent on gap. We assessed the effects of gap on the models using three different ATT values of 100, 500, and 1500 ms. The physiologic and imaging parameters used in the simulation were as following: T_1,a_ = 1664 ms [35], T_2,a_ = 120 ms [36], M_0,b_ = 1, labeling duration = 2 s, flip angle = 60°, and α = 1.

Monte Carlo simulations were performed to validate the bSSFP model by comparing it to a full solution of Bloch equations under different noise levels. In order to describe the real circumstance, each dataset was produced by Bloch equation simulations as described in [37]. Monte Carlo datasets were generated for F values ranging from 100 to 300 ml/min/100ml at an interval of 50 ml/min/100ml. For each flow value, the δ values also ranged from 200 ms to 1000 ms with 200-ms step. The gap was 3 cm, and the velocity of arteries (*v*) varied from 6 to 15 cm/s with 3-cm/s step. Considering vessel geometry, the angle of vessel to the z-direction (φ) varied from 0° to 80° with 20° step. The ATT value was defined by the gap divided by the effective flow (v_z_) in z-direction: *v*_*z*_ = *v* ∙ *cosθ*. For each combination of three parameters (F, δ, and ATT), random noise with Gaussian distribution was added to the data, with SNR of the peak signal increasing from 5 to 20 in intervals of 5. Each data was fitted to the bSSFP model, and then the three parameters were estimated. For each noise level, the procedure was repeated 1000 times to obtain the mean and standard deviation (SD) of the estimated parameters. In order to evaluate the accuracy and precision of the bSSFP model for estimating the three parameters (F, δ, and ATT), the error percentage of the mean and the corresponding coefficient of variation (fitted SD/fitted mean × 100%) were respectively calculated at each noise level [28, 38].

#### Labeling effects on the superior sagittal sinus

In ALADDIN with multiphase bSSFP, both labeling from tissue and labeling from vein can be observed in SSS, but they may show different temporal characteristics as mentioned above. Flip angle is a significant factor affecting the transient and steady-state magnetizations in bSSFP [39]. Thus, simulations with multiple FA values (a range of 30° to 60°) were performed. Inter-slice TD and gap in ALADDIN affect the time interval between labeling in a prior slice and its detection in a subsequent slice. Thus we performed simulations with various TDs of 0, 250, and 500 ms and various gap sizes of 200, 300, 400, and 500% of slice thickness. The effects of labeling from tissue originate from the steady state tissue magnetizations in prior slices which are superior to an imaging slice. In cases of large gap sizes, second or more prior slices are out of brain, and thus only one or two prior slices can act as labeling planes. Thus we approximated the effects of labeling from tissue to be simply in inverse proportion to gap. We computed physiologic parameters of GM for the prediction of the steady state magnetization: T_1,t_ = 1331 ms, T_2,t_ = 80 ms, T_1,v_ = 1584 ms [35], T_2,v_ = 54 ms [36], M_0,b_ = 1, velocity = 10 cm/s, flip angle = 60°, and α = 1. All simulations were implemented in MATLAB (Mathowrks, Natick, MA, USA).

### Ethical considerations

All experiments were approved by the Institutional Review Boards at the Seoul National University and written informed consent was obtained from all participants.

### Experiments

In ALADDIN acquisition, a dataset of four different types of images was acquired: combination of the positive (^+^G_SS_) or negative (-G_SS_) slice-select gradient with the ascending (As) or descending (Ds) acquisition order. Two ascending acquisitions and two descending acquisitions were first averaged individually and then subtracted to maximize perfusion-weighted signals and suppress the MT effects [20, 40]. The readout gradient polarity was also alternated (i.e., total 8 types of images) and then the acquired images were averaged, to suppress eddy current contributions [41].

**Fig 2:**
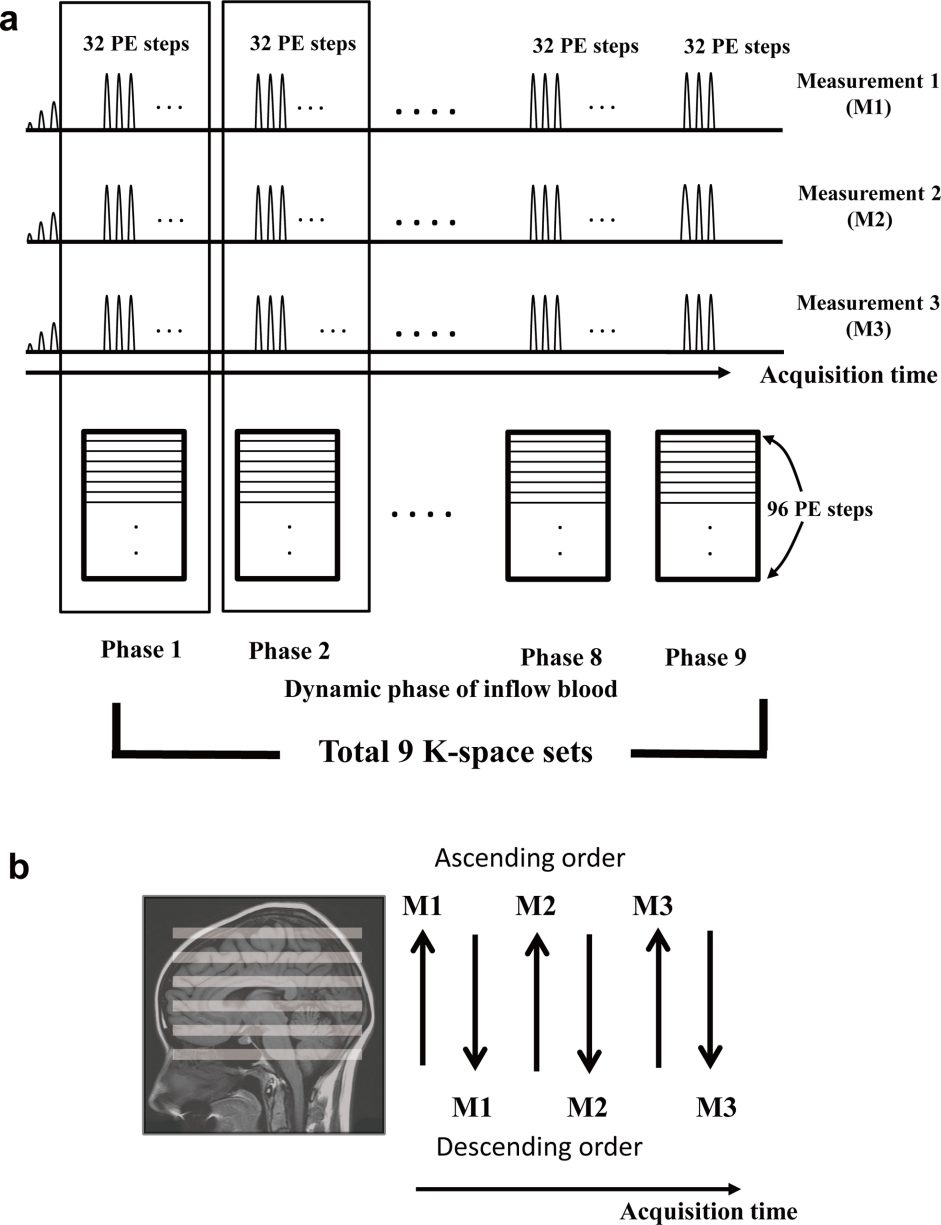
Diagram for segmented multiphase bSSFP sequence. (a) Initial 10 phase encoding scans with linearly increasing flip angles were discarded as dummy scans. Each measurement consisted of 288 phase encoding steps, which were subdivided into nine K-space datasets with 32 phase encoding lines per dataset. Since each K-space reflected different stage of flow dynamics, nine dynamic phases could be obtained. In order to fill 96 phase encoding lines per K-space, three measurements were performed. (b) ALADDIN acquisition scheme was composed of sequential ascending and descending orders, which were alternated in every measurement. Therefore, six measurements were needed to complete nine K-space datasets in both ascending and descending orders.

Dynamic perfusion-weighted data were obtained by combining ALADDIN and multiphase bSSFP acquisition strategy. Initially, a train of 10 dummy RF pulses with linearly-increasing flip angles was applied. Then, data of 288 phase encoding steps per measurement were used to fill nine segmented K-spaces (Fig 2A). Therefore each K-space was filled with 32 phase encoding steps per measurement and three measurements were necessary to completely fill all the 96 phase encoding steps. Six measurements were necessary to fill all K-spaces in both ascending and descending orders (Fig 2B). Through multiphase strategy, dynamic data were obtained with multiple time phases from 108 ms to 1170 ms with an interval of 133 ms.

All experiments were performed on 3T Tim Trio whole body scanners (Siemens Medical Solutions, Erlangen, Germany) with a body coil for transmission and a 12-element head matrix coil for reception. ALADDIN with multiphase bSSFP was performed using flip angle 60° in 6 healthy volunteers. In order to quantify aCBV, data acquisition should start before labeled blood enters capillary compartment and thus large gap was preferred. Total four different sets of experiments with two TDs (0 and 500 ms) and two gap sizes (300 and 450% of slice thickness) were carried out to evaluate effects of these parameters on the quantification of aCBV. For each set of experiments, total 24 measurements were repeated, yielding the 8 different types of datasets per slice with the ALADDIN acquisition strategy. Default imaging parameters to get a set of 2D bSSFP images were: TR / TE = 4.15 / 2.08 ms, matrix size = 128 x 96, FOV = 220 x 220 mm^2^, number of slices = 7−9, acquisition bandwidth = 592 Hz/pixel, phase encoding order = linear, delay time between acquisitions of the 8 types of images = ~1 sec, slice thickness = 8 mm, and total scan time = 4.9−6.5 min (varying depending on TD).

### Data analysis

K-space data was analyzed using in-house program in MATLAB (Mathworks, Natick, MA, USA). In order to minimize MT asymmetry, we compensated for the MT asymmetry effects by acquiring datasets of different slice-selection gradients and acquisition orders. The signal differences ΔS between labeling and control scans was calculated by [20]:

$$\Delta S=(({GssDs}^{-}+{GssDs}^{+})-({GssAs}^{-}+{GssAs}^{+}))/2$$ (8)

To estimate three unknown parameters (ATT, F, δ), dynamic signal differences were analyzed by voxel-wise fitting of the multiphase data to either Eq (2) (T_1_ model) or Eq (5) (bSSFP model). We utilized a nonlinear least-squares algorithm implemented in MATLAB (lsqcurvefit). Arterial CBV was finally computed following Eq (6). The mean F, ATT, δ, and aCBV for each subject were assessed in a region of interest (ROI) of GM using the data from the voxel-wise analysis. ROI covering the whole GM region in the center slice was drawn by a semi-automatic in-house program using the region growing algorithm in MATLAB.

#### ROI analysis for superior sagittal sinus

An ROI covering SSS was manually drawn in a slice which was perpendicular to SSS. Ascending and descending orders respectively corresponded to control and labeling scans in SSS. The 450% gap was equal to the length of 36 mm and thus it took ~280 ms for blood in SSS (velocity = 13 cm/s) to move through the 36-mm gap. This gap was enough to measure labeling from vein during the first two phases (108 ms and 241 ms), as demonstrated later. The signal difference between ascending and descending orders was calculated and then divided by mean signal intensity of the ascending acquisitions to assess the labeling efficiency. In order to verify the two kinds of labeling mechanisms (labeling from vein and labeling from tissue) and evaluate the dependency of labeling effects on gap and TD, data from the experiments with two TD values (0 and 500 ms) and two gap sizes (300% and 450% of the slice thickness) were analyzed.

#### Validation study for measuring aCBV

Although several approaches have been introduced to measure aCBV, there is no consensus about the gold standard. We compared our method with one of the existing methods, termed arterial volume using arterial spin tagging (AVAST) [15], on five healthy subjects to test the feasibility of our method. ALADDIN multiphase bSSFP acquisition was additionally performed with two gap sizes (300 and 450% of slice thickness) and two TD values (0 and 500 ms), followed by AVAST acquisition. Details of AVAST are described in the literature [15]. Briefly, pseudo-continuous tagging pulses were employed with tagging duration varying from 1.0 to 2.0 s with a step size of 0.2 s. Two scans were performed with and without the flow crusher gradient. At first, the tagging duration nulling tissue perfusion signals was found in the images acquired with the crusher gradient. At the tagging duration nulling tissue perfusion signals, ASL signals in scans without the crusher gradient were found and considered related to aCBV. Single slice bSSFP readout was used in the AVAST experiments. An ROI with number of pixels more than 150 was drawn manually in GM of the posterior lobe. Arterial CBV values estimated by the bSSFP and T_1_ models were compared to that of AVAST. Pearson correlation coefficients were evaluated between AVAST and our method on a voxel-by-voxel basis in the whole brain of the center slice, at a reduced resolution (64 × 64 matrix) to minimize effects of potential motion-related displacements.

## Results

### Simulations

#### Quantification of aCBV

**Fig 3:**
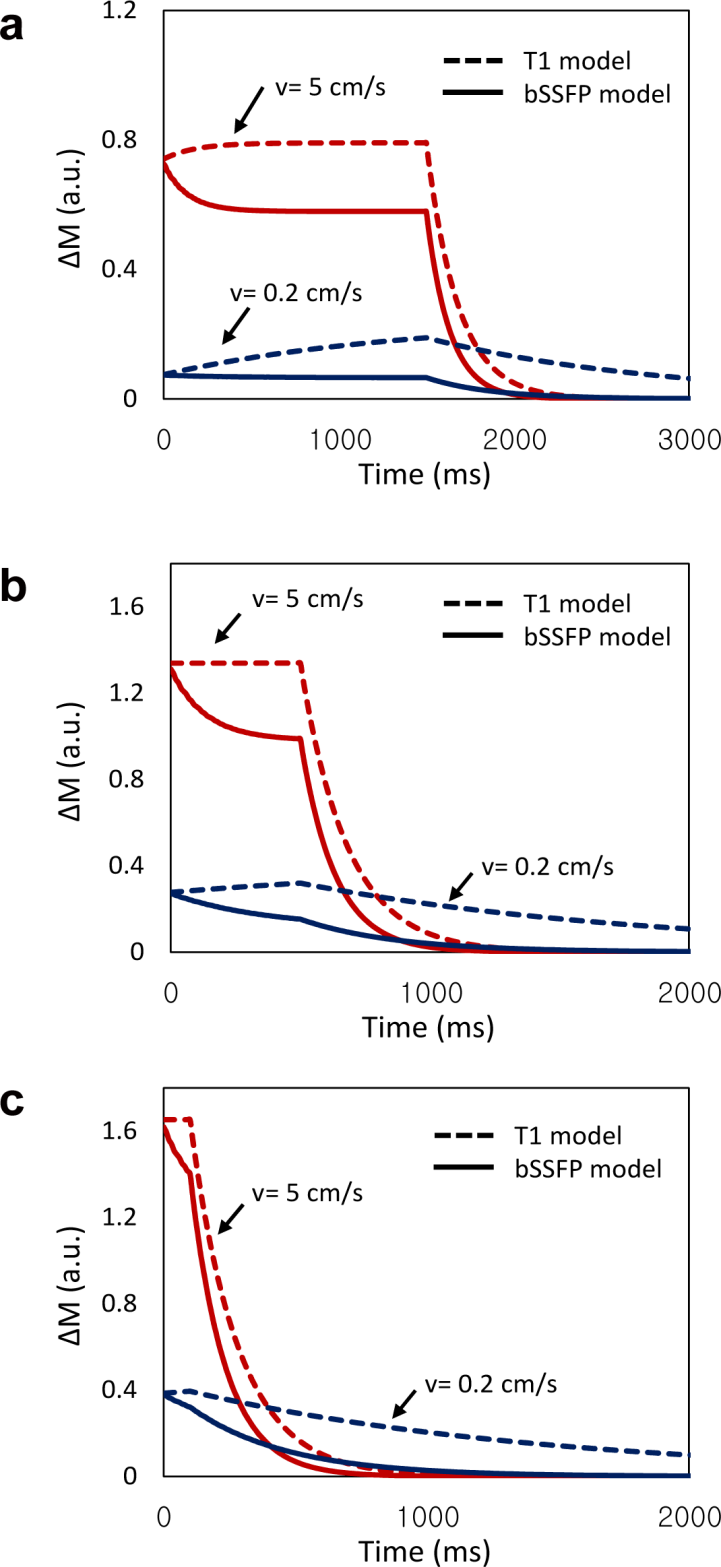
Simulation results of magnetization difference between control and labeling scans with two flow rates (0.2 and 5 cm/s) for ALADDIN with multiphase bSSFP sequence. The simulations were performed at ATT of 1500 ms (a), 500 ms (b), and 100 ms (c). The red and blue lines represent the flow rates of 0.2 cm/s and 5 cm/s, respectively. The solid and broken lines represent the results for fitting to bSSFP model and T_1_ model, respectively. ΔM represents magnetization difference between control and labeling scans. a.u. represents arbitrary unit.

Fig 3 shows results of two models at two different blood velocities. The longitudinal magnetization difference ΔM between labeled and unlabeled spins for slow blood flow (0.2 cm/s) markedly diminished, while those for rapid blood flow (5 cm/s) was less disturbed by RF pulses. The selective loss of ΔM in the slow blood flow was also demonstrated even in cases of short ATT (Fig 3B and 3C), indicating that tissue perfusion was suppressed in multiphase bSSFP readout irrespective of ATT values and thus that we could acquire labeled blood signals predominantly from arterial blood. The difference between the bSSFP model and the T_1_ model reflected the loss of ΔM in capillaries and tissue by RF pulses (Fig 3). When ATT was long, image acquisition could start before labeled capillary blood exchanged with tissue water and thus the loss of ΔM by RF pulses was maximized for a longer period of time (Fig 3A).

**Fig 4:**
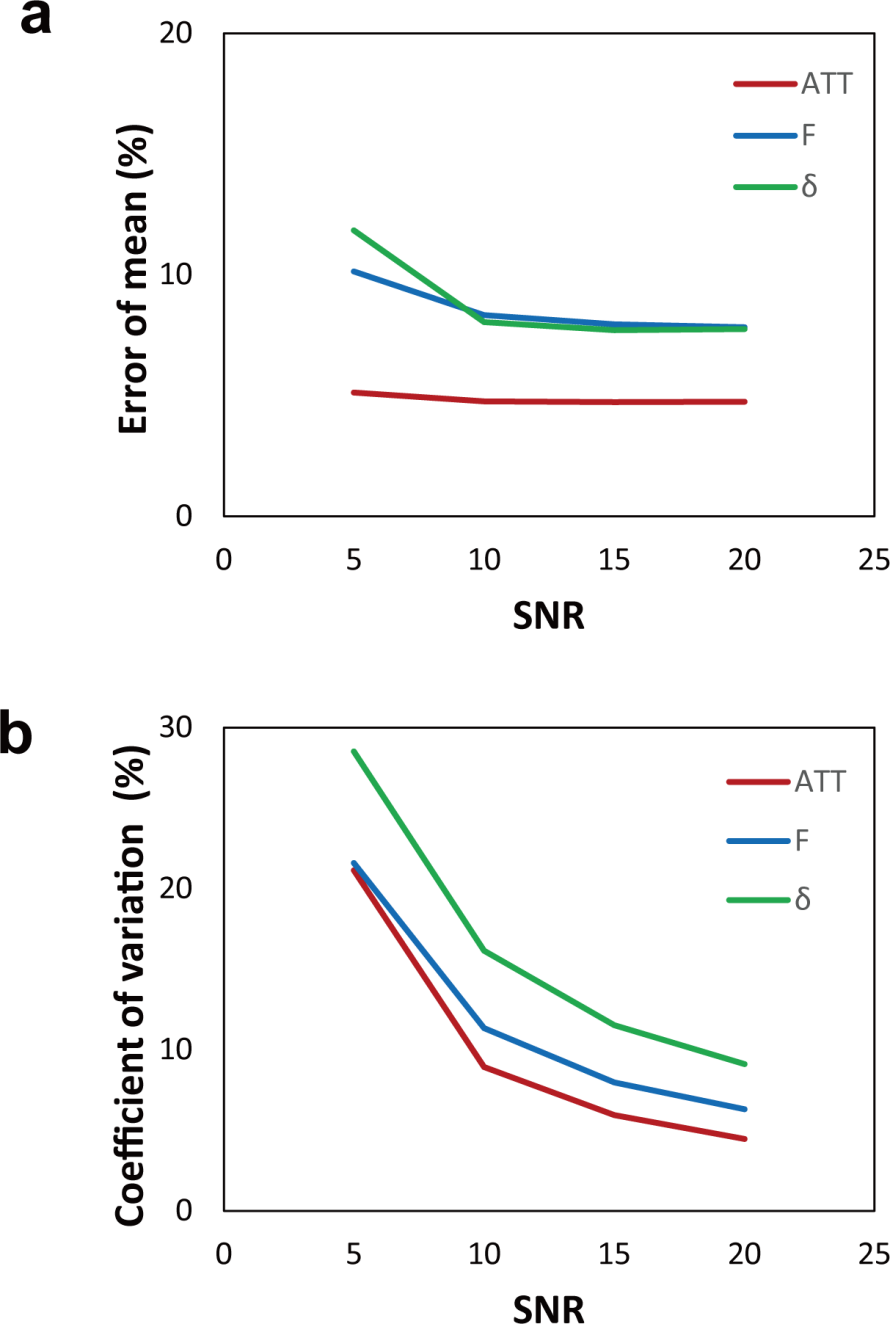
Monte Carlo simulation results for the bSSFP model. The error percentage of the mean (indicating the accuracy) (**a**) and the coefficient of variation (indicating the precision) (**b**) are shown as a function of the signal-to-noise ratio (SNR) that ranged from 5 to 20 with a step size of 5.

Fig 4 shows the results of Monte Carlo simulations. The error of the mean of the three parameters decreased with SNR (Fig 4A), and the accuracy of the three parameters (ATT, F, and δ) at SNR = 20 were 4.5, 6.3, and 9.1, respectively. At the same SNR level, the fitted ATT values showed higher accuracy. The coefficient of variation of three parameters decreased with SNR (Fig 4B). These results were similar to the results of the previous Monte Carlo simulation study for the quantification of cerebral blood flow [28], indicating feasibility of our model (Eq (5)) for aCBV quantification.

#### Labeling effects on the superior sagittal sinus

**Fig 5:**
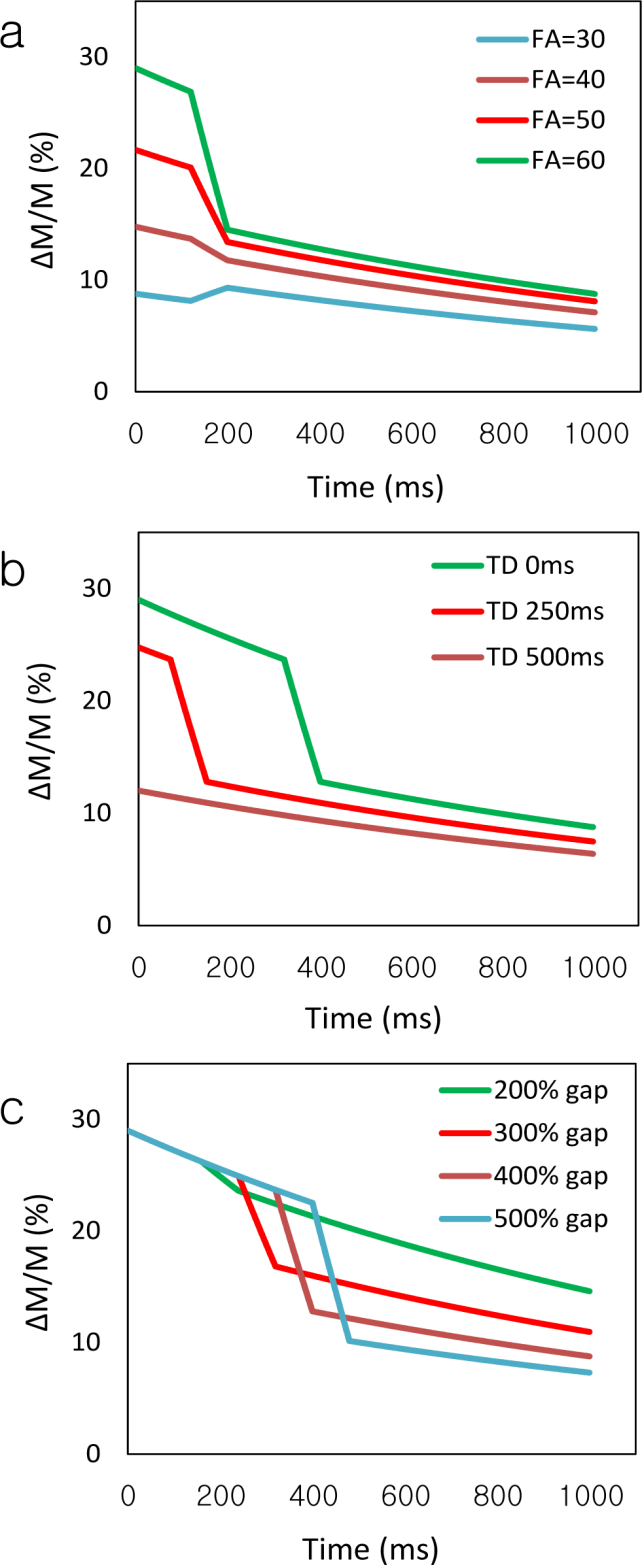
Simulation results of magnetization difference between control and labeling scans in sagittal sinus vein. The simulations were performed at various flip angles (FA) (30, 40, 50, and 60°) (a), various inter-slice delay time (TD) (0, 250, and 500 ms) (b), and various gap size (200, 300, 400, and 500% of slice thickness) (c). ΔM represents magnetization difference between control and labeling scans and M represents the signal intensity of control scan.

Fig 5 shows the rapid decay of ΔM at the earlier phases and the slow decay at the later phases in SSS. Labeling from vein and labeling from tissue generally increased with FA (Fig 5A). At FA 60°, two different effects became prominent; labeling from vein at the early phases and labeling from tissue at the later phases. Since blood affected by labeling from vein rapidly moved out of the imaging slice, it was not detected in 500-ms TD (Fig 5B). The labeling from vein was detected for a longer time at 500% gap due to increased ATT (Fig 5C), supporting the idea that large gap is needed to detect labeling from vein. In all cases, labeling from tissue was persistently demonstrated until the later stage, because the acquisition time of total nine phases (~1.2s) was shorter than T_1_ relaxation time (1664 ms) [35].

### Experiments

#### Quantification of aCBV

**Fig 6:**
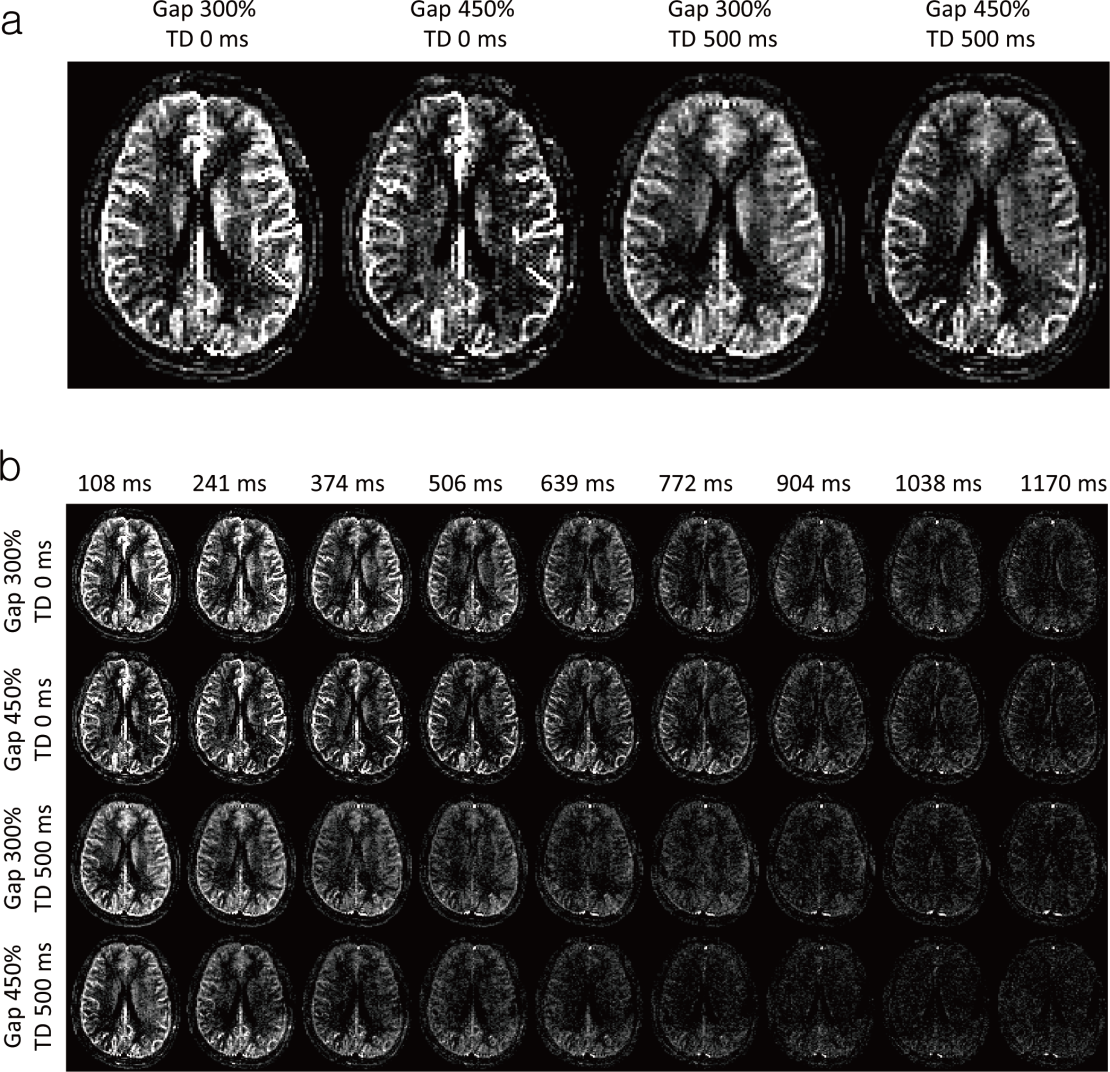
A representative dataset of multiphase ALADDIN with different gap sizes (300% and 450% of slice thickness) and different inter-slice delay time (TD) (0 and 500 ms) from one representative subject. (a) The enlarged perfusion-weighted images at the first phase. (b) The perfusion-weighted images from the multiple phases. The time phase of perfusion-weighted images is marked on top of each column. Scan time for 0-ms TD and 500-ms TD was 4.9 min and 6.5 min, respectively.

A representative dataset of multiphase ALADDIN perfusion-weighted imaging is displayed in Fig 6. The experiments with two gap sizes and two TDs demonstrated various perfusion-weighted images in the first phase. In the first phase images (Fig 6A), arterial signals were stronger with shorter TD (0 ms) and larger gap (450%), whereas tissue perfusion signals were apparent with longer TD (500 ms) and smaller gap (300%). With progress of temporal phases, arterial signals decayed slowly while tissue signals decayed faster. Strong tissue perfusion signals in the first phase image with 500-ms TD (lower two rows in leftmost column, Fig 6B) were not observed in the later phase images (time phase of 506 ms) with TD 0 ms (upper two rows in 4^th^ left column, Fig 6B), supporting the notion that tissue perfusion signals were significantly perturbed by RF pulses as predicted in Fig 3A. These results showed that both gap and TD affect the arterial vascular tree detected in aCBV.

**Fig 7:**
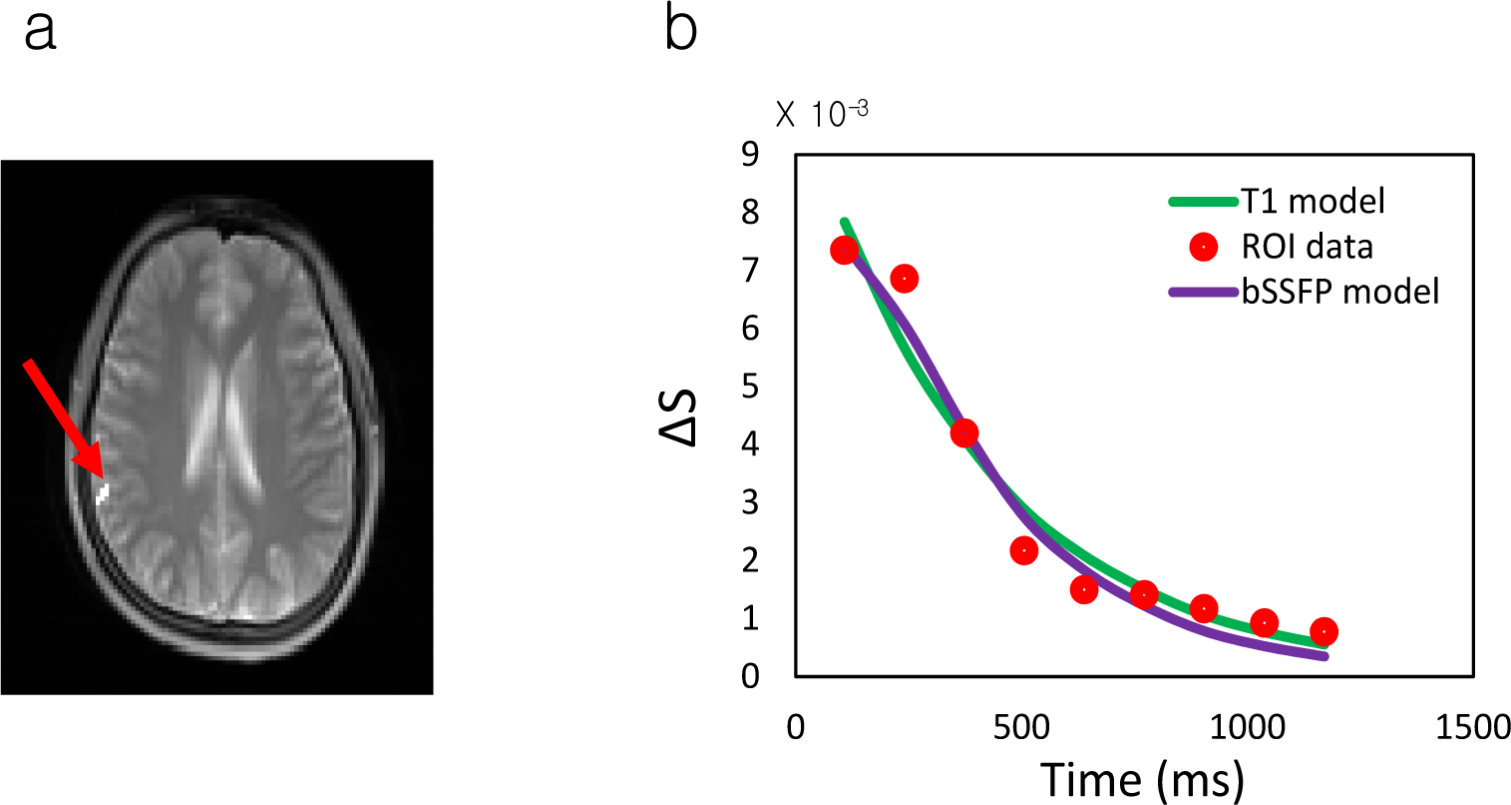
Representative results of fitting to the proposed models. (a) Location of region of interest (ROI) which is drawn manually (red arrow). (b) Dynamic signal difference in the ROI shown in (a) and results of curve fitting with T_1_ model (green line) and bSSFP model (purple line). The data was acquired with 450% gap and 0-ms inter-slice time delay. ΔS represents signal difference between control and labeling scans.

A representative data for curve fitting of sampled data to the models in GM ROI is shown in Fig 7. Fitted results were similar to those of simulations with 100-ms ATT (Fig 5C). Table 1 shows estimated F, δ, and ATT from voxel-by-voxel analysis. ATT values increased with gap, as expected. The ATT values in bSSFP model (628−748 ms in gap of 24−36 mm) were in good agreement with those from literature (ATT = 401 ms in 17-mm gap and 607 ms in 45-mm gap) [8, 42]. The δ values (589–661 ms) were similar to the transit time in arterial compartment reported in literature (390–570 ms) [8, 43]. The F values were lower in 500-ms TD than those in 0-ms TD, presumably due to the fact that most labeled blood spins passed the arterial compartment during the delay time of 500 ms and tissue perfusion signals were suppressed by RF pulses during multiphase acquisition. The ATT and δ values from the T_1_ model were lower than the bSSFP model (Table 1). The difference between the two models was presumably related to the different decay rates of the two models, as shown in the simulations (Fig 5).

**Table 1:** The estimated values from two proposed models. Perfusion parameters. ATT: traveling time from a labeling plane to an imaging slice, F: arterial flow parameter, δ: transit time from the arterial compartment to the capillary/tissue compartment in imaging slice. All values represented in the form of Mean ± SD* were measured in gray matter from ALADDIN with multiphase bSSFP readout fitted to bSSFP model and T_1_ model.

		ATT (ms)		F (mL/100 mL/min)		δ (ms)	
Gap	TD	bSSFP	T_1_	bSSFP	T_1_	bSSFP	T_1_
**450%**	0 ms	628 ± 118	484 ± 100	197 ± 37	140 ± 20	661 ± 34	427 ± 25
	500 ms	673 ± 158	379 ± 110	139 ± 29	136 ± 21	609 ± 48	418 ± 28
**300%**	0 ms	748 ± 174	530 ± 135	191 ± 51	162 ± 22	609 ± 75	374 ± 55
	500 ms	647 ± 221	378 ± 104	132 ± 29	127 ± 31	589 ± 48	391 ± 37

* Mean values were calculated for each subject first and then averaged to get the inter-subject mean (Mean) and standard deviation (SD).

**Fig 8:**
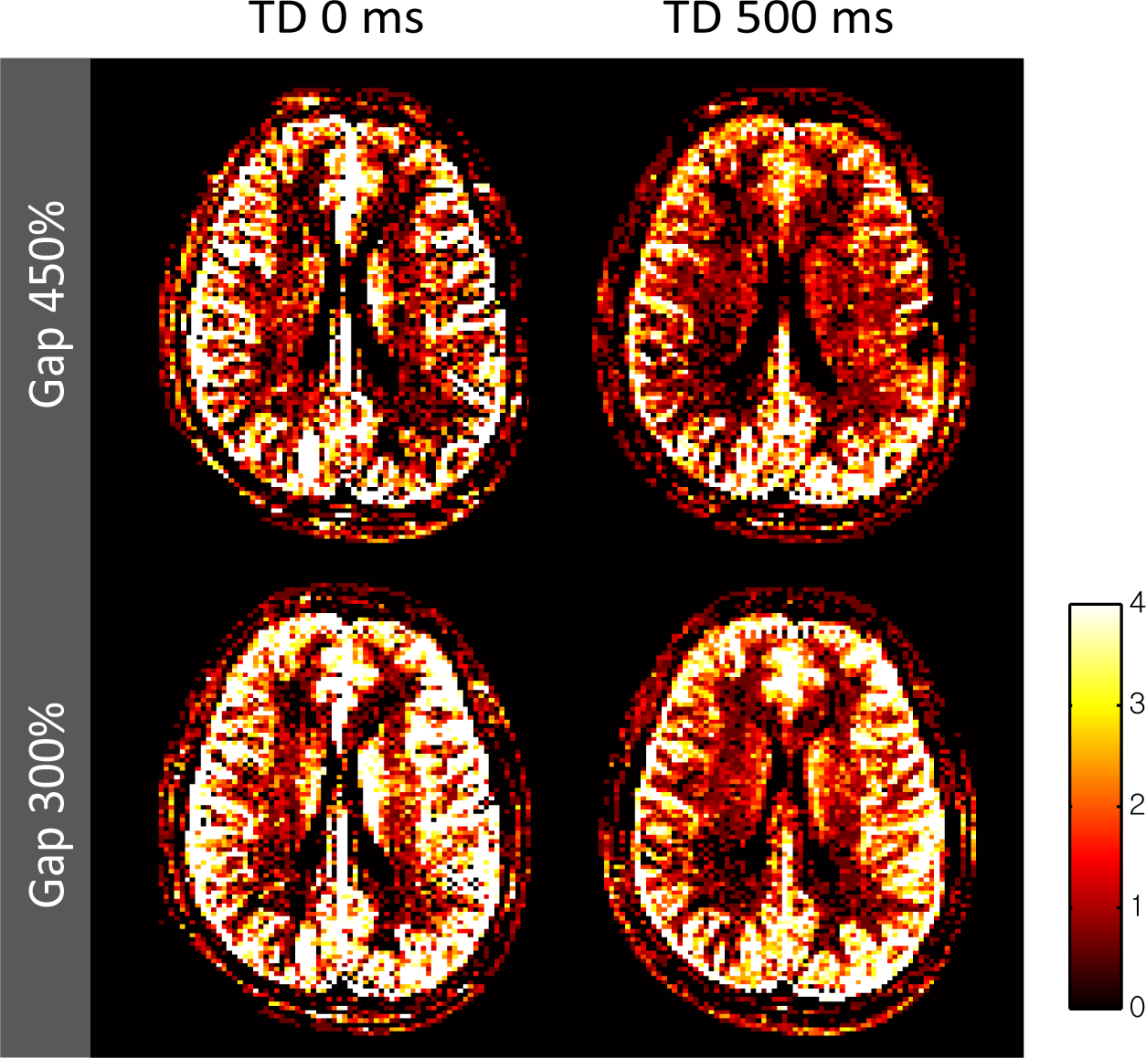
Representative arterial cerebral blood volume (aCBV) maps from the same subject as shown in Fig 6. The values in the color scale bar are displayed in the unit of mL/100 mL.

Table 2 shows the estimated aCBV values in GM (1.4–2.3 mL/100 mL), which were in good agreement with the published data in the range of 0.76−2.80 mL/100 mL [5, 8, 9, 16, 33]. The highest aCBV value was measured at the experiment with 300% gap and 0-ms TD, which might be caused by short travel distance and maximized labeled signals in the arterial compartment due to short TD. Fig 8 showed a representative set of aCBV maps. Signals from large arteries were definitely shown at 0-ms TD, while those were suppressed in aCBV map at 500-ms TD. The average aCBV of T_1_ model was smaller than that of bSSFP model for all subjects (Table 2).

**Table 2:** Comparison of estimated arterial cerebral blood volumes (aCBVs) (Mean ± SD*) between bSSFP model and T_1_ model (N = 6).

Gap	TD	bSSFP	T_1_
**450%**	0 ms	1.93 ± 0.54	1.13 ± 0.31
	500 ms	1.41 ± 0.49	0.79 ± 0.26
**300%**	0 ms	2.31 ± 0.75	1.30 ± 0.38
	500 ms	1.41 ± 0.59	0.77 ± 0.29

* unit for aCBV: mL/100 mL

* Mean values were calculated for each subject first and then averaged to get the inter-subject mean (Mean) and standard deviation (SD).

#### Labeling effects on the superior sagittal sinus

**Fig 9:**
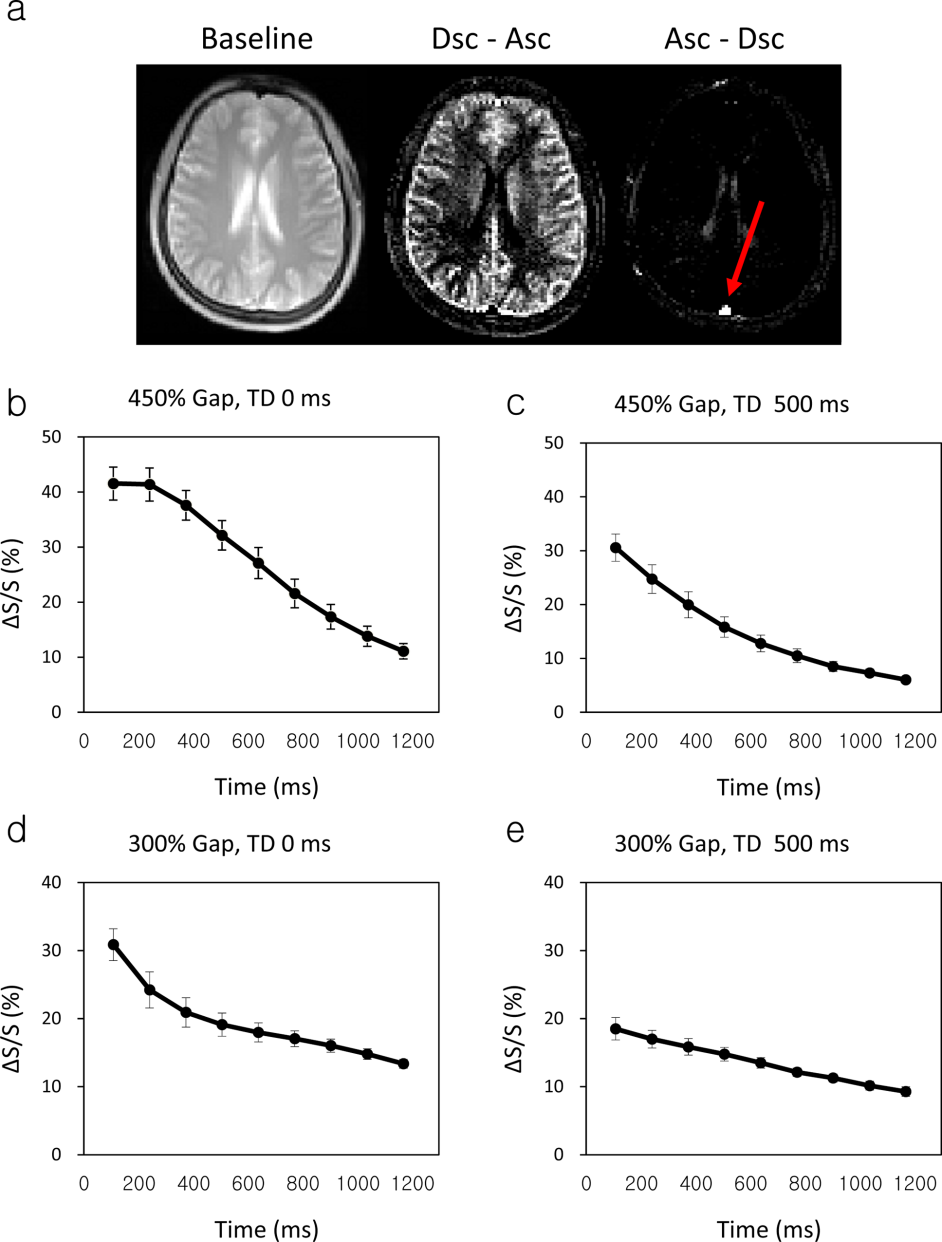
ROI analysis in superior sagittal sinus (SSS). (a) Images of baseline, labeling effects from feet to head (Des − Asc), and labeling effects from head to feet (Asc − Dsc) from a representative slice used for measurement of SSS perfusion signals. (b)−(e) Multiphase signal differences between labeling and control scans in SSS measured from all the 6 subjects for inter-slice gap sizes of 450% (b,c) and 300% (d,e) and inter-slice delay time (TD) of 0 ms (b,d) and 500 ms (c,e). Vertical bars represent the standard deviation across the 6 subjects.

A representative perfusion-weighted image showing descending flow is displayed in Fig 9A, and the ROI analyses in SSS are shown in Fig 9B–9D. Descending flow in lateral ventricles was incidentally detected, presumably due to turbulent flow in the apex of the lateral ventricles. Maximum labeling effects (20.8±3.7%) were detected in SSS with plateau during the initial 2 phases in scans with 450% gap (Fig 9B). The maximum labeling effects could be considered as the labeling efficiency (α) of ALADDIN technique, and this value was used in curve fitting of dynamic data. As expected in simulations, the signal difference between labeling and control scans decreased rapidly during the early phase(s) of scan and were slowly reduced in the middle and late phases of scan (Fig 9D). Labeling from vein was not observed at 300% gap and 500-ms TD (Fig 9E), presumably due to the fact that blood affected by labeling from vein had already passed the slice. The labeling from tissue was not completely eliminated until the last phase of scan, because of relatively slow decay rate, which is dependent on T_1_ relaxation time (1664 ms) [35]. These experimental observations were in good agreement with the simulation results (Fig 5).

#### Validation of measuring aCBV

**Fig 10:**
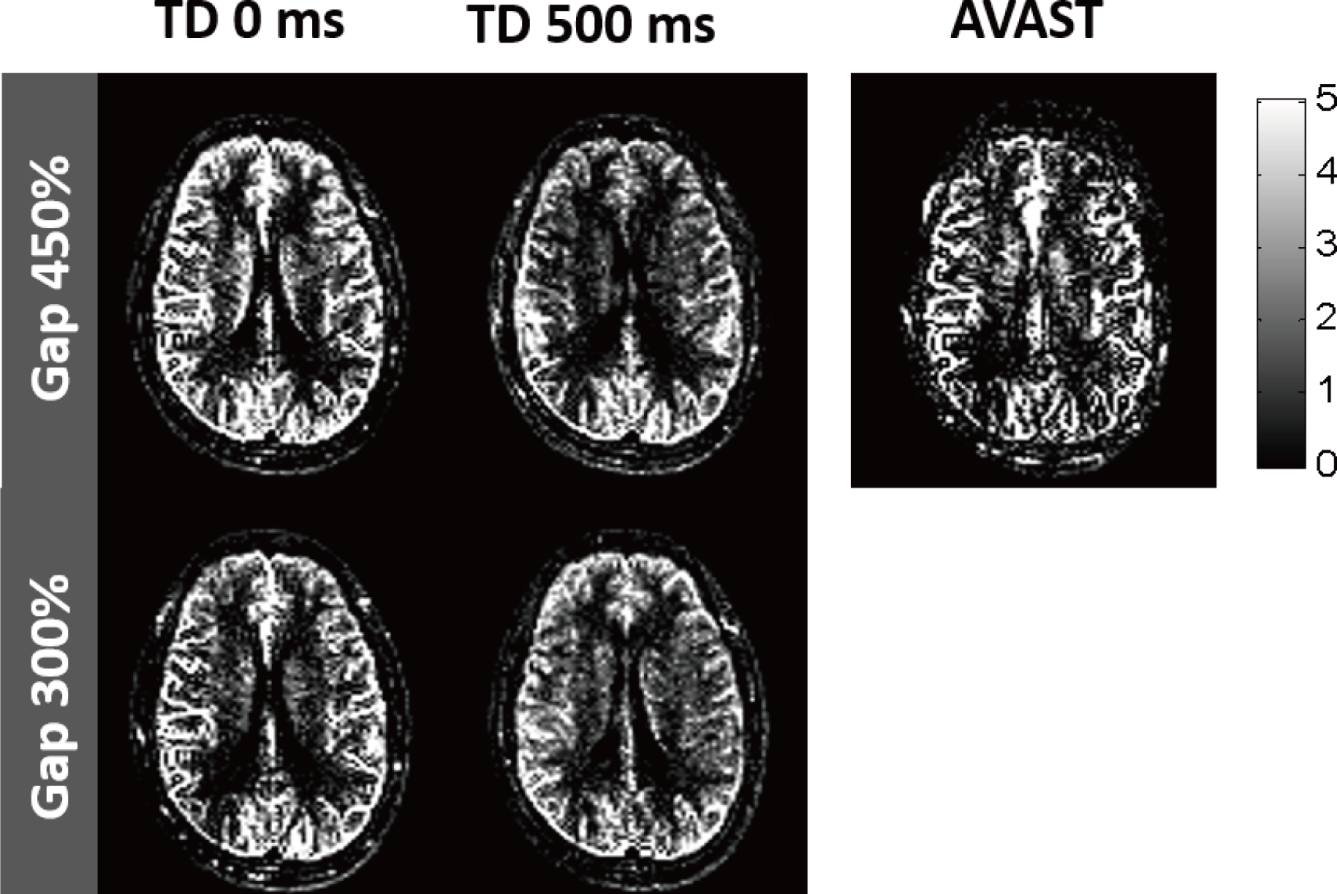
Representative aCBV maps of ALADDIN with multiphase bSSFP and AVAST. Estimated aCBV maps of ALADDIN with four different conditions and AVAST are shown, and the values in the gray scale bar are displayed in the unit of mL/100 mL.

Table 3 shows aCBV values from the proposed ALADDIN multiphase bSSFP and AVAST, and representative aCBV maps are shown in Fig 10. The measured aCBV values from the proposed method were similar to those from AVAST. AVAST generally showed stronger signals in large arteries, while the proposed method showed stronger signals both in large arteries and tissue regions, indicating the higher sensitivity of the proposed method to smaller arterial branches. There was positive correlation (P < 0.05) between our technique and AVAST across all subjects with higher correlation at 0-ms TD; r = 0.53 ± 0.07 at 300% gap and 0-ms TD, r = 0.53 ± 0.07 at 450% gap and 0-ms TD, r = 0.22 ± 0.07 at 300% gap and 500-ms TD, and r = 0.32 ± 0.04 at 450% gap and 500-ms TD.

**Table 3:** Comparison of estimated arterial cerebral blood volumes (aCBVs) (Mean ± SD*) between multiphase bSSFP and AVAST (N = 5).

Gap	TD	bSSFP	T_1_	AVAST
**450%**	0 ms	2.17 ± 0.88	1.27 ± 0.54	
	500 ms	1.87 ± 0.67	1.01 ± 0.32	2.43 ± 0.37
**300%**	0 ms	2.12 ± 0.87	1.22 ± 0.51	
	500 ms	1.85 ± 0.88	1.01 ± 0.43	

* unit for aCBV: mL/100 mL

* Mean values were calculated for each subject first and then averaged to get the inter-subject mean (Mean) and standard deviation (SD).

## Discussion

Our study presents the feasibility of quantifying aCBV using ALADDIN with multiphase bSSFP readout. As rapidly flowing blood is less disturbed by a long train of RF pulses, ALADDIN with multiphase bSSFP readout allows to selectively emphasize arterial blood. By fitting the multiphase data to the proposed bSSFP model, three unknown parameters (ATT, F, and δ) were estimated. Arterial CBV values in GM calculated from the measured F and δ values (1.41–2.31 mL/100 mL) were in good agreement with those from literature, and comparison studies of our method and AVAST method also showed similar results.

A couple of changes were made to the conventional ASL model to represent the ALADDIN-based aCBV signals better. First, arterial input function in the form of *unit*(*ATT−t*) was adopted in this study to reflect the labeling characteristics of ALADDIN. Second, a new model modified from the general kinetic model was proposed in consideration of the bSSFP RF perturbations [27, 28]. Signal decay in multiphase bSSFP acquisition is different from that in conventional ASL techniques that follows T_1_ relaxation, because multiphase bSSFP readout drives blood spins to the steady state condition [30]. There were differences in the measured values between the bSSFP model and the T_1_ model (Table 2). Although the T_1_ model is simpler, the bSSFP model is expected to better reflect the loss of labeling effects by the long RF pulse train.

When the bSSFP model was used for curve fitting analysis, ATT and δ were well correlated with those from the literature, while arterial flow parameter F (Table 1) was significantly higher than the tissue perfusion value from literature (50−60 mL/100 g/min) [44–46]. When one artery supplies a certain territory, the flow measured in the arterial compartment (in the unit of blood volume per unit time) should remain the same as the flow measured in the tissue compartment (mean CBF × total tissue volume supplied by the artery) [47]. However, arterial flow per unit volume (as measured in this study as the parameter F) may be higher than the CBF values, because of smaller total volumes in the arterial compartment than the tissue compartment supplied by the artery.

Labeled blood measured with multiphase ALADDIN may originate not only from macro- and micro-vasculature in a voxel, but also from large arteries that pass through the voxel. Also tissue perfusion signals may contribute to the measured blood signals. Such complicated contributions can lead to potential overestimation of aCBV. In order to suppress contamination from large arteries, crusher gradient is sometimes used. A recent study using iVASO technique has reported that aCBV value falls from 2.04 to 0.76 with application of a bipolar gradient [8]. This approach is reasonable as the crusher gradient eliminates the contributions from arteries moving faster than a certain threshold velocity. However, an optimal cut-off value, which classifies the arteries into two types of supplier and passerby, has not been defined yet. Thus, further study should be performed to confirm that the gradient crusher can select only arteries supplying regional tissue.

In addition to the contribution of large arteries, labeled blood in capillaries and tissue can contribute to the labeled blood signals. Although multiphase bSSFP readout decreases the labeled blood signals in capillary and tissue quickly as shown in the simulations (Fig 5) and experiments (Fig 6), the early phase images can include tissue perfusion signals and thus F values may be overestimated and δ values may be underestimated. In order to suppress such contamination, large gap should be applied to increase the traveling time from labeling planes to an imaging slice. Since aCBV is calculated by multiplying δ and F, however, the potential errors in F and δ may be cancelled out and thus the contribution of tissue perfusion to aCBV may be small.

In order to optimize ALADDIN with multiphase bSSFP readout for imaging of aCBV, labeled blood signals in the arterial compartment should be maximized and tissue perfusion should be minimized. Flip angle is an important parameter affecting the labeling efficiency induced by inter-slice blood flow effects and also affecting the loss of labeling effects by a long train of RF pulses. High flip angle has two advantages; (i) maximizing inter-slice blood flow effects, which increase labeled blood signals in both arterial and tissue compartments, and (ii) suppressing the labeled blood signals in the tissue compartment during multiphase bSSFP readout [16]. Thus, high flip angle is desired within the acceptable level of RF energy tissue deposition. In this study, 60° flip angle was used to generate sufficient interslice labeling effects. Although 40° flip angle was recommended in the previous aCBV study using multiphase bSSFP because of RF power deposition [16], specific absorption rate was acceptable in our experiments by using a slightly longer RF duration of 1.2 ms.

Inter-slice Gap and TD are also important to suppress tissue perfusion signals. With long TD, arterial signals decreased while tissue perfusion increased in early phase(s), and thus aCBV can be contaminated by tissue perfusion signals. Also, wide gap is preferred to measure arterial signals, as demonstrated in experiments with 450% gap where arterial signals were dominant. Therefore, wide gap and short TD are preferred for quantifying aCBV.

As mentioned above, flip angle, inter-slice gap, TD, and also slice thickness can affect the vascular tree that is really assessed with the proposed method. As long as the labeled blood spin is in the arterial compartment during the transit time δ, it can be considered moving faster than 13 mm/sec in arteries with diameter ≥20 μm [18]. When slice thickness = 8 mm, the number of RF pulses experienced by the blood spin is less than 150, at which the signal decay is ~50% according to our previous simulation study [20]. Therefore, arterial vessels with diameter ≥ 20 μm will maintain the ASL signals more than 50%, whereas those with diameter < 20 μm will have much lower signals. This sensitivity of the proposed method to the small arterioles agrees with the results shown in Fig 10.

ALADDIN has additional advantage of visualizing bi-directional flow effects (ascending/descending) in single experiment [40]. In ALADDIN, labeling mechanisms in SSS reflect two effects; (i) labeling from vein due to inter-slice blood flow effects, (ii) labeling from tissue originating from the steady state magnetization of tissue. These effects may provide information about venous drainage tract or brain tissue. If total obstruction of SSS or global tissue impairment such as large territory infarction occurs, the dynamics in SSS is expected to be changed. However, further study is necessary in order to justify its clinical roles in detecting pathology.

Our models described flow dynamics of labeled blood traveling through the vascular structure, replacing complicated simulations using the Bloch equations. In previous literature, the approach using a convolution function is proved equivalent to that of the Bloch equations in the kinetic models for ASL signals [27, 29]. The decay by RF pulses in bSSFP [30, 37] can be simply added to the kinetic models and replace T_1_ relaxation term, without solving the Bloch equations. Also we found in our previous studies that the Bloch equation simulations and the simple bSSFP equations provide the same pattern of perfusion signal decay by RF pulses in bSSFP readout [21, 24]. Therefore, we proposed the simple and straightforward models derived by a convolution function instead of using the Bloch equations.

Labeling efficiency of ALADDIN was smaller than that of conventional ASL techniques in both simulations in the previous literature [21] and experimental studies in this work, although we take into account the potential underestimation of labeling efficiency in this work (measurement as percent signal changes in SSS). In ALADDIN, however, time delay from labeling to imaging is shorter (typically < 0.5 sec) because of shorter traveling distance from labeling plane to imaging slice and labeling duration is longer (typically > 3 s) than that of the conventional ASL methods. In pseudo-continuous ASL (pCASL) post labeling delay (PLD) of ~1.5 sec and labeling duration of ~1.5 sec are typically used. Therefore, low labeling efficiency could be considerably compensated by the advantage of short post labeling delay and long labeling duration in ALADDIN.

The long scan time for whole brain coverage is one limitation of the proposed approach. For example, multiphase ALADDIN with 300% gap and TD 0-ms should be performed three times in a spatially interleaved manner to cover whole brain, and total acquisition time is about ~15 min. As all ASL techniques are sensitive to motion artifacts because of subtraction between labeling and control scans, the proposed method is also expected to be sensitive to motion. The current ALADDIN technique with multiphase bSSFP seems to provide predominantly aCBV-weighted images, however, capillary and tissue signals may also contribute to aCBV signals. As this method depends on the number of RF pulses that the labeled arterial and capillary bloods experience in the imaging slice, pathology affecting flow rate can lead to over–or underestimation of aCBV. Therefore, the scan parameters may need to be selected depending on the vascular trees of interest specific to disease types. Arteries running within the imaging slice can also lead to underestimation of aCBV, because of increased number of RF excitations. Finally, labeling mechanism of ALADDIN can be affected by effective velocity in slice selection direction, which determines the number of RF pulses that blood spins experience in a prior slice. To influence signals in a subsequent slice, labeled blood from prior slices should pass through the large gap (2.4 and 3.6 cm) quickly at 500-ms TD. Thus mean effective velocity should be high enough to contribute to the ASL signals in the subsequent slice. Previous simulations showed that the labeling efficiency did not change significantly within the effective velocity range of artery in ALADDIN, when off-resonance flow spins and the spins of even and odd numbers of RF excitations were averaged [20]. Therefore the labeling efficiency of ALADDIN is considered to be less sensitive to vascular orientation or velocity.

In conclusion, we presented a new ASL technique for quantifying aCBV without intravascular contrast agent injection. Multiphase acquisition strategy was implemented in ALADDIN to acquire dynamic data of labeled blood signals in both the arterial compartment and SSS. Using the kinetic model, the dynamic data was analyzed to estimate unknown parameters (F, δ, ATT) on voxel-by-voxel basis. The estimated aCBV values were comparable to those from an existing method (AVAST) and generally in agreement with the reported values from literature. This approach can be an alternative to existing techniques for the quantification of aCBV. Labeled blood in SSS provided not only the information of the labeling efficiency, but also physiologic information of drainage of tissue blood into SSS, which could be potentially helpful for clinical research and neuroscience applications.
